# 

**Published:** 1880-04

**Authors:** 


### HYMENEAL.

"Domestic happiness, thou only bliss
Of paradise that has survived the fall."

Hopkins—Roberts.—In Philadelphia, on the 28th of Second month, 1880, by Friends' ceremony, Dr. Charles E. Hopkins and Hannah W., daughter of Elihu Roberts. All of that city.

---

### NATALITIAL.

"Of all the joys that brighten suffering earth,
What joy is welcomed like a new-born child."

Born, in Norfolk, Virginia, March 16, 1880, a son to Dr. Harvey L. and Mrs. Florence Byrd.

### SPECIAL NOTICE.

From among a large number of letters commendatory of THE INDEPENDENT PRACTITIONER, we take pleasure in making excerpts from a few from different sections of our country, indicative of the estimate of our labors thus far.

PROF. ISAAC E. TAYLOR, M. D., President of Bellevue Medical College, New York, says: "I have been much pleased with the matter introduced, as well as the dress of the Journal, and I wish you all success in the effort to advance the interests of the profession. I noticed a favorable criticism in the *Medical Record* of last week." A few weeks later he gave us a valuable paper which is published in our February issue.

PROF. ROBERTS BARTHOLOW, M. D., of the Jefferson Medical College, Philadelphia, says: "I trust the Journal is succeeding and that you find your editorial work congenial. I shall be pleased to send you a paper when I get through the work which I am compelled to do."

PROF. J. M. DA COSTA, M. D., of the same college and city, writes: "I wish it (the Journal) a most marked success."

PROF. THOS. H. CHANDLER, Dean of the Harvard University, Dental Department, writes: "I am well pleased with your first number. If you keep up the standard, your magazine will fill a void in our professional literature."

PROF. F. PEYRE PORCHER, M. D., Charleston, S. C., writes: "I have read your Journal with pleasure."

PROF. MIDDLETON MICHEL, M. D., of the same city and college, says: "I am much pleased with the appearance and contents of your Journal and wish it success."

PROF. JAS. E. GARRETSON, M. D., D. D. S., Dean of the Philadelphia Dental College, writes: "Let me wish you success in an enterprise which looks as if calculated to be of great use to the dentist. I have read each number of the Journal with much satisfaction and have to apologize for my tardiness in acknowledging your kindness in sending it. I welcome the 'PRACTITIONER' particularly from the standpoint of its *medico*-dental character. The more dentists learn of general medicine and surgery the better are they found qualified for work as specialists."

PROF. J. C. LE HARDY, M. D., of Savannah, Ga., writes: "I am very much pleased with the 'INDEPENDENT PRACTITIONER.'"

PROF. V. H. TALIAFERRO, M. D., of Atlanta, Ga., says: "I will cheerfully contribute my mite to aid in your effort to continue the Journal a grand success."

PROF. W. H. DOUGHTY, M. D., Augusta, Ga., "I wish your enterprise abundant success."

COL. J. J. WOODWARD, Surgeon U. S. Army, "I wish for your enterprise a useful and honorable career and all success."

DR. B. P. ANDERSON, President of the Medical Society of the State of Colorado: "Your Journal is a perfect success. It will give me a great deal of pleasure to assist the 'INDEPENDENT PRACTITIONER' in any way I can."

PROF. WM. A. HAMMOND, M. D., N. Y., and formerly Surgeon-General of the U. S. Army, says: "I am pleased with the 'INDEPENDENT PRACTITIONER,' and wish you success."

PROF. ISAAC J. WETHERBEE, D. D. S., President of the Boston Dental College, writes: "A careful examination of the contents of the 'INDEPENDENT PRACTITIONER' leads me to believe that it will be a very useful publication to the dental profession, and hope that you may meet with large success."

DR. W. R. MUNROE, of Baltimore, "I am happy to receive the 'INDEPENDENT PRACTITIONER,' so neatly and beautifully published, and so ably edited by yourself and colleague. Your prospectus is striking and convincing as to the propriety of your independent position, and I wish you great success in the enterprise you have so nobly inaugurated."

Others among the most intelligent members of the profession in this city have verbally expressed similarly encouraging words.

DR. B. W. HARDEE, of Savannah, Ga., "Merit must and will meet with success."

The foregoing extracts have been taken almost at random from a large number of letters, now before us, and pages could be filled with similar expressions from them.

Space will allow of the mention of a few more names only, of those who have sent us happy greetings or promised articles, viz.: PROF. WM. PEPPER, M. D., PROF. S. WEIR MITCHELL, M. D., DR. RICHARD J. DUNGLINSON, PROF. CHAS. J. ESSIG, D. D. S, M. D., Philadelphia; PROF. T. GAILLARD THOMAS, M. D., DR. GEO. M. BEARD, and others, of New York, etc.

We hazard nothing in saying that no medical journal started in this country has been more generally commended, verbally and otherwise, by readers entitled to respect and confidence.

***Our Column of "Paid-up" Subscribers. 1880 to 1881.***

[To be continued.]

Geo. M. Beard, M. D., N. Y. City.
Prof. Jas. E. Garretson, M. D., D. D. S., Phila., Pa.
Dr. B. Dozier, Cal.
Dr. Wm. A. Byrd, Ill.
Prof. C. F. W. Bodecker, D. D. S., M. D. S., N. Y. City.
Prof. Isaac E. Taylor, M. D., N. Y. City.
Dr. C. T. Stockwell, Mass.
Prof. Jas. H. Harris, M. D., D. D. S., Balt.
Dr. A. C. Ford, Fla.
Dr. S. E. Jones, Texas.
Dr. Edw. Maynard, Wash.
Dr. Geo. McCowan, Cal.
Dr. B. H. Teague, S. C.
Dr. C. E. Francis, N. Y. City.
Dr. C. Millett Babb, Maine.
Dr. W. H. Berthelot, La.
Dr. Thos. Brian Gunning, N. Y. City.
Dr. J. M. Tevey, Va.
Dr. W. W. Wilkerson, Ala.
Prof. Laurence Turnbull, M. D., Phila.
Dr. B. P. Anderson, Col.
Dr. C. C. Dills, Conn.
Dr. C. A. Wilkerson, Ala.
Prof. Isaac J. Wetherbee, D. D. S., Boston.
Dr. Jno. C. Uhler, Balt.
Dr. J. C. Hanks, N. J.
Dr. Fred. Koerner, Balt.
Dr. W. S. Alsop, Va.
Dr. P. P. Palmer, S. C.
Dr. Chris. Fawcett, Balt.
Dr. Wm. H. Hoopes, Balt.
Dr. W. M. Wilkerson, Ala.
Dr. Walter S. Harban, Wash.
Drs. E. W. and B. L. Lane, Ga.
Dr. R. P. Greenleaf, Dela.
Dr. Thos. H. Davy, Balt.
Dr. P. H. Brown, Jr., Conn.
Dr. J. B. Wood, Va.
Library Surgeon-General's Office, War Department, Washington.
Dr. J. A. Anderson, N. Y. City.
Dr. Evan Norton, S. C.
Drs. S. C. and J. C. Wilkerson, Ala.
Dr. Thos. W. Reed, Mo.
Dr. G. Liebman, Balt.
Dr. J. A. Osman, N. J.
Dr. G. H. Chewning, Va.
Dr. H. B. Noble, Wash.
Dr. R. W. Morgan, Va.
Dr. Geo. Phelps, Ga.
Dr. Chas. H. Richards, Dela.
Dr. J. D. Twyman, Ky.
Dr. Cyrus See, Pa.
Prof. Middleton Michel, M. D., S. C.
Prof. Frank Abbott, M. D., N. Y. City.
Dr. S. A. Beecher, Minn.
Dr. Bryan F. Coy, Balt.
Dr. D. M. Culver, Ind.
Dr. Rufus W. Griswold, Conn.
Dr. Robt. B. S. Hargis, Fla.
Dr. Geo. E. Matthews, N. C.
Dr. Benj. F. Rea, Ala.

This column will be sold for advertising space six months during the year on *special rates*.

N. B.—We invite attention to the names in our "paid-up" column on this page, also to "Special Notice" in this journal. We hope those who have not sent in their subscription money will now find it convenient and agreeable to do so.

